# HIV Prevention Among People Who Inject Drugs (PWID): A Narrative Review

**DOI:** 10.7759/cureus.85719

**Published:** 2025-06-10

**Authors:** Amanda Matter, Sawsan Younan

**Affiliations:** 1 Pharmacy, Massachusetts College of Pharmacy and Health Sciences, Boston, USA; 2 Family Medicine, Trillium Health Partners, Mississauga, CAN

**Keywords:** harm reduction, hiv prevention, integrated care model, medication adherence strategies, opioid agonist therapy (oat), pre-exposure prophylaxis (prep), pwids (people who inject drugs), stigma in health care, syringe service program

## Abstract

People who inject drugs (PWID) face a significantly higher risk of HIV infection than the general population. Effective prevention for this group depends on combining several strategies: needle and syringe programs (NSPs), opioid agonist therapy (OAT), antiretroviral therapy (ART), pre-exposure prophylaxis (PrEP), overdose prevention, and changes to structural and policy environments. This narrative review draws on evidence published between 2000 and 2024, including systematic reviews, clinical trials, surveillance reports, and global health guidelines.

Programs that offer high coverage of NSPs and OAT have consistently been shown to reduce HIV incidence. ART helps lower viral load and prevents transmission, while both daily and long-acting PrEP offer additional protection for those at risk. However, access remains limited, particularly in low- and middle-income countries, due to criminalization, stigma, and a lack of investment in harm reduction services.

Outcomes vary widely by region. In places like Portugal and Canada, where policies support decriminalization and integrate harm reduction into routine care, HIV incidence among PWID has dropped significantly. In contrast, countries that maintain punitive drug laws, such as Russia, continue to struggle with sustained epidemics.

Reducing HIV transmission in this population will require expanding access to proven interventions, integrating services more effectively, and reforming policies that act as barriers to care. Priorities moving forward include scaling up NSP and OAT, addressing gender-based inequities, reducing stigma, and aligning national policies with international harm reduction standards.

## Introduction and background

People who inject drugs (PWID) are at a higher risk of HIV infection. Globally, an estimated 11 million individuals inject drugs, with 12-15% living with HIV; prevalence varies widely by region [1-3]. Rates often surpass those observed in the general population, reaching over 30% in parts of Eastern Europe and Central Asia. In Latin America and Southeast Asia, prevalence tends to be lower but remains a significant concern [1,3]. In Thailand, for example, the estimated prevalence among PWID was 7.8% in 2020, with limited surveillance data available from neighboring countries such as Cambodia and Laos [3].

A range of structural and policy factors contribute to this elevated risk. Criminalization of drug use, stigma, incarceration, housing instability, and gender-based discrimination limit access to essential health services [4]. These barriers not only affect individual health but also contribute to ongoing transmission through shared injection equipment, sexual contact, and perinatal exposure [5-7].

Combination prevention, as defined by UNAIDS, involves using biomedical, behavioral, and structural strategies in tandem to reduce HIV transmission [4,8]. High-coverage needle and syringe programs (NSPs) and opioid agonist therapy (OAT) are among the most effective biomedical strategies for PWID [9,10]. When delivered alongside HIV testing, antiretroviral therapy (ART), and pre-exposure prophylaxis (PrEP), these interventions can significantly reduce incidence [11,12].

This review synthesizes global evidence on biomedical HIV prevention strategies for PWID, including NSPs, OAT, PrEP, and ART, while examining structural barriers, demographic disparities, and legal and policy environments that influence service access.

Despite strong evidence supporting these interventions, implementation remains limited. Fewer than one percent of PWID live in countries that meet WHO targets for both NSP and OAT coverage [3,7,13]. Access is particularly constrained in low- and middle-income countries and among marginalized groups such as women and LGBTQ+ individuals, who often face multiple levels of exclusion [8-10]. A 2023 global report found that 40 percent of PWID experienced stigma in healthcare settings in the past year, and 17 percent avoided care as a result [1,4].

This review further emphasizes the need for improved integration of harm reduction into national health systems, guided by evidence, equity considerations, and the removal of legal and structural barriers to care.

## Review

Methods

Review Design and Reporting

We conducted a comprehensive narrative review of the literature on HIV prevention strategies for PWID. The review adhered to key principles of the PRISMA 2020 guidelines, adapted appropriately for a narrative review. Although a formal review protocol was not registered, the search strategy and study selection process were predefined to enhance methodological transparency.

Literature Search and Selection

We systematically searched four electronic databases: PubMed/MEDLINE, Embase, Web of Science, and the Cochrane Library. The search included literature published from January 2000 through December 2024. It employed a combination of controlled vocabulary terms and free-text keywords related to HIV prevention and injection drug use. Key terms included “HIV prevention,” “people who inject drugs,” “PWID,” “harm reduction,” “needle and syringe programs,” “opioid substitution therapy,” “pre-exposure prophylaxis,” and “structural interventions.” To supplement database searches, we manually screened the reference lists of key articles, studies, and recent systematic reviews to identify additional relevant publications.

Inclusion and Exclusion Criteria

We included studies of any design that addressed HIV prevention interventions or outcomes among PWID populations. Eligible study types encompassed randomized controlled trials, observational studies, modeling analyses, qualitative research, systematic reviews, and authoritative policy reports. Included studies were required to report outcomes related to HIV incidence, HIV prevalence, risk behaviors, or the coverage and uptake of prevention interventions. Only articles published in English were considered. Studies were excluded if they did not focus specifically on PWID or HIV prevention, lacked sufficient methodological detail, or were classified as editorials, opinion pieces, case reports, or conference abstracts.

Study Selection and Data Extraction

Two reviewers independently screened the titles and abstracts of all records identified in the search. Full-text articles of potentially eligible studies were then reviewed in detail by both reviewers. Discrepancies regarding study inclusion were resolved through discussion and consensus. One reviewer extracted relevant data from the included studies, and a second reviewer independently verified the extracted data to ensure accuracy and consistency. Given the narrative nature of this review, a PRISMA flow diagram was not generated, and no formal meta-analysis or quantitative synthesis was conducted.

Key interventions

Effective HIV prevention for PWID requires a combination of interventions that address both biological and behavioral risks. Core strategies include OAT, NSPs, PrEP, and ART, delivered as part of treatment as prevention (TasP). Additional approaches, such as supervised injection facilities (SIFs), naloxone distribution for overdose prevention, and integrated service models, help reduce high-risk behaviors, improve access to care, and support sustained engagement. Although each intervention has demonstrated benefit independently, the greatest impact is achieved when they are implemented together through coordinated, accessible programs.

Needle and Syringe Programs

NSPs provide sterile injection equipment and safe disposal, which reduces syringe sharing and related transmission risks. The World Health Organization (WHO) defines high-coverage NSPs as distributing at least 200 syringes per PWID annually [14,15]. Programs that meet this target are associated with reductions in HIV incidence of approximately 50 percent [6,16]. Many NSPs also offer HIV testing, hepatitis screening, condom distribution, and safer sex counseling. When co-located with HIV care or primary health services, NSPs serve as single-access points that enhance program reach. In well-established programs, sustained high coverage has been linked to significant declines in HIV incidence among PWID [5,6,16].

In many regions, NSP availability remains limited due to restrictive policies and criminalization. In some countries, syringe possession is penalized, which discourages individuals from seeking services. Between 2016 and 2021, the global median distribution was estimated at fewer than 40 syringes per person per year, which falls well below WHO recommendations [3]. Coverage is especially low in Asia, Africa, and Latin America. In Eastern Europe and Central Asia, the median is closer to 20 syringes per year [7,12]. NSPs are rarely available in prison systems, where risk is high and access is especially limited.

Opioid Agonist Therapy

Medications such as methadone and buprenorphine lower the frequency of injection and reduce overdose risk. Programs reaching at least 40 percent of PWID are considered high-coverage by WHO and UNAIDS, and reaching this threshold has been associated with significant reductions in HIV transmission [3,17-19]. Globally, only 10 to 20 percent of PWID have access to OAT, with coverage generally higher in Western Europe and lower in most low- and middle-income countries [1,7,14]. Both methadone and buprenorphine are effective in reducing risk when patients remain in treatment [19].

Access to OAT is often restricted by national policies and regulatory barriers. In many countries, OAT medications are banned or tightly controlled [14]. Criminalization of drug use discourages individuals from seeking treatment. Incarcerated PWID frequently lack access to OAT, despite a high risk of disease [14]. Women, migrants, and other underserved populations face additional access challenges. In countries where access has expanded, outcomes have improved. For example, Portugal's decriminalization policies in 2001 were followed by increased OAT enrollment and a decline in injection-related HIV transmission [15,20].

Combined NSPs and OAT

Modeling studies suggest that scaling up NSPs and OAT simultaneously could reduce HIV incidence among PWID by more than 70 percent [5,19]. However, only a few regions, including parts of Australia and Western Europe, meet WHO coverage targets for both services [15]. Globally, fewer than one percent of PWID live in areas where both interventions are implemented at recommended levels [7,14]. This service gap remains a significant barrier to HIV control.

Pre-exposure Prophylaxis

Daily oral PrEP provides additional protection for PWID at risk of HIV. The Bangkok Tenofovir Study found a 49 percent reduction in HIV incidence among participants taking PrEP, with over 70 percent efficacy among those with adequate adherence [21,22]. WHO and the U.S. Centers for Disease Control and Prevention recommend PrEP for PWID who are at ongoing risk [14]. Despite this, uptake remains low due to a lack of awareness, stigma, and access challenges [23-25].

Newer long-acting PrEP formulations, such as injectable cabotegravir, have shown approximately 96 percent efficacy in trials when compared to daily oral PrEP [26]. Although these options may improve adherence, data specific to PWID are limited. Pilot studies suggest that embedding PrEP delivery into NSP and OAT services may help increase uptake [27]. PEP is also an option, but evidence on its use among PWID is sparse. Increasing access to both PrEP and PEP will require policy reform, improved affordability, and reduction of criminalization-related barriers.

HIV Testing and ART

Early HIV testing and prompt ART initiation improve health outcomes and reduce transmission through TasP [28]. When viral load is suppressed to undetectable levels, transmission risk is nearly eliminated. Access to testing and ART among PWID varies greatly. Some urban centers report strong engagement, while rural areas and low-income settings experience limited coverage [1,29].

Outreach and integration strategies have improved outcomes in certain settings. In Vancouver, integrated programs helped increase viral suppression rates among PWID [30-32]. In contrast, many U.S. regions continue to report low ART uptake and adherence in this population [1,31]. Incarcerated individuals are often left out of HIV treatment programs. ART access in prisons frequently falls below 10 percent, and the absence of OAT and NSP further undermines prevention efforts [14,21]. Test-and-treat approaches designed for PWID will be more effective if delivered alongside comprehensive harm reduction services [29].

Overdose Prevention (Naloxone)

Opioid overdose is a major cause of death among PWID [2]. Distribution of naloxone and training in overdose response can reduce mortality and support continued care [2]. Although naloxone does not directly prevent HIV transmission, it plays an essential role in keeping individuals alive and linked to healthcare services.

Access to naloxone varies by region. Community-based distribution is legal in many high-income countries, but policy and cost barriers persist elsewhere [2,14]. The WHO recommends making naloxone widely available as part of a comprehensive harm reduction package [14]. Programs that integrate overdose prevention with HIV and addiction services improve both survival and care retention.

Supervised Injection Facilities (SIFs)

SIFs are licensed facilities where PWID can inject pre-obtained substances under professional supervision using sterile equipment. These programs are associated with reductions in syringe sharing, overdose mortality, and public injection [30-32]. SIFs also serve as entry points to healthcare. Evaluations report that SIF clients are more likely to be referred to HIV and addiction treatment and are less likely to engage in behaviors that increase transmission risk [33,34].

Despite this evidence, SIFs remain limited to a small number of high-income countries including Canada, parts of Europe, and Australia [15]. Legal and political barriers have prevented their adoption in most low- and middle-income countries. Pilot projects in several cities have demonstrated feasibility. Broader implementation will require legal reform and policy support.

Integrated Service Models

Programs that integrate HIV prevention and treatment services are more effective at engaging PWID and improving health outcomes. Models that coordinate HIV testing, NSPs, OAT, PrEP, and case management report higher rates of retention, adherence, and viral suppression [13,35]. In some studies, one-stop service models achieved viral suppression rates several times higher than those of conventional care systems [35,36].

These models often rely on peer outreach, streamlined intake processes, and co-located services to minimize barriers to care. Despite promising outcomes, integrated programs remain uncommon. Program fragmentation, siloed funding streams, and restrictive regulations continue to limit implementation. Supporting integrated care through shared funding, flexible policy frameworks, and interagency coordination will be critical to expanding access for PWID.

**Table 1:** Overview of Core HIV Prevention Strategies for People Who Inject Drugs: Effectiveness and Implementation Considerations NSP: Needle and Syringe Programs; associated with approximately 50% reduction in HIV incidence through reduced syringe sharing; OAT: Opioid agonist therapy; linked to 50–70% reduction in HIV incidence and improved retention in care; ART: Antiretroviral therapy; critical for achieving viral suppression and preventing onward HIV transmission when initiated early; TasP: Treatment as Prevention; refers to ART’s role in reducing HIV transmission at the population level; PrEP: Pre-exposure prophylaxis; shown to reduce HIV incidence by 49% in a randomized trial among PWID, with greater efficacy among adherent individuals; PWID: People Who Inject Drugs; SIFs: Supervised injection facilities. SIFs are legally sanctioned sites that reduce high risk injection behaviors and improve linkage to care; Integrated service models: Co-located or coordinated models of care that improve ART outcomes and service engagement among PWID; Overdose prevention (Naloxone): Naloxone distribution and overdose response training reduce opioid-related mortality and help maintain engagement in care among PWID. Source: [5-7,12,16,18,25,28,30,32,33,35,37,38]

Intervention	Evidence Type	Summary of Outcomes and Impact
Needle and Syringe Programs (NSPs)	Systematic reviews; mathematical modeling; observational studies	High-coverage NSPs (≥200 syringes per PWID per year) are associated with approximately 50% reduction in HIV incidence. Integrated programs that combine NSP with testing and primary care may further enhance impact. Global NSP coverage remains below target levels.
Opioid Agonist Therapy (OAT)	Systematic reviews; randomized controlled trials (RCTs); observational studies	Achieving ≥40% OAT coverage is linked to substantial reductions in HIV transmission (50–70%). Methadone generally achieves higher treatment retention than buprenorphine. Global OAT coverage remains low, particularly in correctional settings and among marginalized populations.
Combined NSP and OAT	Mathematical modeling; observational studies	Combined scale-up of NSP and OAT is projected to reduce HIV transmission by over 70%. Currently, fewer than 1% of PWID live in areas where both interventions meet recommended targets.
Pre-Exposure Prophylaxis (PrEP)	Randomized controlled trial (Bangkok Tenofovir Study); observational studies	Oral PrEP demonstrated 49% reduction in HIV incidence among PWID in an RCT, with ≥70% efficacy among those with detectable drug levels. Real-world uptake remains low due to stigma and access barriers; acceptability is moderate to high. Long-acting injectable PrEP is promising but untested in large-scale PWID studies.
Antiretroviral Therapy (ART)	Randomized controlled trials; meta-analyses; observational studies	Early ART initiation and viral suppression reduce HIV transmission (treatment-as-prevention). ART access and adherence are lower among women, incarcerated PWID, and unstably housed individuals. Integrated outreach models have improved ART outcomes in some settings.
Overdose Prevention (Naloxone)	Program evaluations; observational studies	Naloxone distribution and overdose response training are highly effective in reducing opioid-related mortality. While indirect, overdose prevention helps maintain engagement in care among PWID.
Supervised Injection Facilities (SIFs)	Cohort studies; program evaluations	SIFs are associated with reduced syringe sharing and public injection, increased referrals to addiction treatment, and reductions in overdose mortality. Their availability remains limited globally.
Integrated Service Models	Observational studies; program evaluations	Integrated models (co-located or closely coordinated services) have improved ART retention and viral suppression. "One-stop" clinics have achieved three-fold higher viral suppression compared to standard care. Peer-led models enhance service engagement.

Regional and demographic variations

Access to HIV prevention varies widely by region. Countries like Canada, Australia, and those in Western Europe distribute over 40 sterile syringes per PWID annually and maintain 30 to 50 percent OAT coverage [7]. These efforts have contributed to historically low HIV incidence. In contrast, most low- and middle-income countries offer minimal access to harm reduction services [7,37,38].

In Eastern Europe, Central Asia, the Middle East, and much of Africa and Asia, NSP and OAT coverage remains critically low [7,37]. Russia, where OAT is banned and NSP access is limited, reports HIV prevalence exceeding 20 percent among PWID, with some areas nearing 60 percent [36].

Women who inject drugs face added barriers stemming from gender-based stigma, fear of child custody loss, and intimate partner violence [1,9,13]. LGBTQ+ individuals experience layered discrimination related to both drug use and minority status, which further limits access to care [10].

An intersectional approach is essential. For instance, a transgender woman who injects drugs, lacks stable housing, and has been incarcerated may be excluded from services across multiple sectors, including criminal justice, housing, and healthcare [38-40]. Addressing these gaps requires not only peer-led engagement but also policy and resource alignment. In Indonesia, community networks improved HIV testing and ART linkage by embedding outreach in local systems [41]. In Canada, peer-run shelters aligned with municipal health mandates have improved access to harm reduction [36].

Unless national programs prioritize marginalized subgroups and embed services into local infrastructure, these disparities will continue to undermine prevention efforts.

Social and structural barriers

National drug policy frameworks strongly shape HIV prevention outcomes. Countries that have adopted decriminalization, such as Portugal, Canada, and parts of Western Europe, typically provide legal, publicly funded harm reduction services [20,42]. In contrast, many low-income countries retain punitive laws and lack institutional support for these interventions [30].

Portugal illustrates the long-term impact of policy reform. After decriminalizing drug use in 2001, HIV cases linked to injection dropped from over 1,200 to just 16 by 2019 [1,20]. However, fewer than 20 percent of countries have implemented WHO and UNAIDS harm reduction guidelines [3].

Stigma remains a major barrier. PWID who experience discrimination are more likely to delay or avoid prevention and treatment [1,43]. Most prison systems lack access to OAT, NSP, or ART, despite substantial documented need among incarcerated populations [21,22].

Housing instability also undermines continuity of care. Individuals without secure housing are less likely to maintain OAT or achieve viral suppression on ART [44-46]. These breakdowns weaken broader prevention strategies. Meta-analyses indicate that more than one-quarter of PWID still share syringes, a pattern that reflects both service fragmentation and insufficient public accountability [47].

Closing these gaps will require coordinated national strategies that integrate healthcare, criminal justice, and housing systems, with funding mechanisms designed to support sustained implementation.

Case studies 

Global examples show both progress and setbacks in HIV prevention among PWID. In Vancouver, Canada, a coordinated harm reduction approach, including expansion of NSPs and the 2003 opening of Insite, North America's first supervised injection facility, led to major public health improvements [32]. Syringe sharing fell from approximately 40 percent in 1996 to under 2 percent by 2011 [20,30,34], and overdose deaths declined in neighborhoods near the facility [32]. By 2012, HIV incidence among PWID had dropped to fewer than two cases per 100 person-years [34].

Portugal achieved similar success through policy reform. After decriminalizing drug use in 2001, the country saw a sharp decline in injection-related HIV diagnoses, highlighting the impact of supportive laws combined with comprehensive care models aligned with public health mandates [20].

In contrast, Russia faces one of the world’s most severe HIV epidemics among PWID. The ongoing ban on OAT, limited syringe access, and punitive policing have contributed to uncontrolled transmission [10,37]. This case underscores how criminalization and service exclusion fuel persistent risk despite clear evidence of effective alternatives.

Outbreak responses and lessons

Recent HIV outbreaks have emphasized the need for robust prevention infrastructure and rapid response systems. In rural Indiana, a 2015 outbreak resulted in 181 new HIV and hepatitis C infections among PWID [31,46]. The public health response included distributing sterile syringes, expanding OAT access, and intensifying contact tracing. Modeling suggests that earlier implementation of NSPs could have prevented much of the spread [5,18].

A similar outbreak in Dublin between 2014 and 2015 was driven by increased stimulant injection and homelessness. The response included scaling up NSP coverage, outreach-based testing, and rapid ART initiation through coordinated public health units [12].

In Athens, Greece, the economic crisis between 2011 and 2013 coincided with a 15-fold rise in HIV incidence among PWID. Expanded harm reduction services, including NSPs, OAT, and intensified testing, reduced incidence by 89 percent within two years [48]. This scale-up was supported through national health insurance and NGO collaboration, illustrating the importance of institutional readiness.

These examples show that fragile infrastructure and policy delays heighten outbreak risk. However, when governments and health systems respond decisively, even large-scale transmission can be reversed. Sustained investment, cross-sector governance, and rapid harm reduction deployment should be embedded into national preparedness strategies. Integrating harm reduction into core public health systems, rather than treating it as emergency response, is key to long-term epidemic control.

Discussion

Despite the availability of highly effective HIV prevention strategies, global implementation remains uneven, particularly for PWID. NSPs, OAT, PrEP, and ART all demonstrate strong individual-level benefits, and their combined use is associated with meaningful reductions in HIV incidence [14,15,18,21]. Still, fewer than 1 percent of PWID globally live in places where both NSP and OAT are offered at recommended levels [5,7]. Structural, legal, and systemic barriers continue to limit progress.

Punitive drug laws remain one of the most persistent obstacles. In many countries, even carrying sterile injection equipment or participating in OAT can lead to arrest or police harassment [4,20,22]. These laws discourage people from seeking services, especially in areas with heavy surveillance. Incarceration also interrupts care, and the period after release is associated with heightened risk of overdose and HIV transmission [21,27]. International guidelines from WHO and UNAIDS recommend offering OAT and NSP in prisons, yet coverage remains extremely limited [14,21,31].

Stigma adds another layer of exclusion. Many PWID report avoiding healthcare altogether due to fear of judgment, discrimination, or criminalization [1,4,5]. For women, these barriers are often compounded by caregiving responsibilities, gender-based violence, or fear of losing custody [8,9,26]. Transgender and LGBTQ+ individuals face additional challenges accessing culturally safe or affirming services [3,10,15].

While many programs emphasize individual behavior change, structural support is just as important. Housing stability, decriminalization, and co-located services all play a role in improving retention and outcomes. Peer navigation and case management programs have helped reduce system-level barriers in some settings [24,29], but these models remain underutilized, especially in low- and middle-income countries.

Global progress also varies dramatically by region. Countries like Portugal and Canada have demonstrated how legal and structural reform can improve access and outcomes [20,26]. But in many other regions, particularly Eastern Europe, Central Asia, the Middle East, and sub-Saharan Africa, punitive laws and underinvestment in harm reduction remain the norm [2,12,13]. National policy reviews show ongoing gaps in meeting WHO’s recommended HIV prevention package, especially where donor support has declined or domestic funding is insufficient [31].

Test-and-treat programs designed for PWID have shown promise by reducing transmission through TasP [28]. However, their success depends on consistent access to ART, OAT, and PrEP. In many settings, these services remain siloed, and viral suppression rates stay low when care is not coordinated [30].

Despite a growing evidence base, important knowledge gaps persist. Most implementation studies come from high-income countries, while regions with the greatest need are underrepresented [2,3]. Few studies report outcomes by gender identity, incarceration status, or migration experience, making it difficult to design inclusive interventions [5,13,15,29].

Bridging the prevention gap for PWID requires more than expanding biomedical tools. It calls for systemic change. Global health funding has prioritized clinical interventions over legal reform or social investment, contributing to uneven harm reduction access [14, 20]. WHO and UNAIDS have emphasized the importance of integrated, rights-based care models-but policy alignment and resource commitments remain inconsistent [8,14,31]. Biomedical tools alone cannot overcome systems built to exclude. Without legal reform, structural protection, and sustained investment in integrated services, the global goal of reducing HIV transmission among PWID will remain out of reach.

Limitations

This review has several limitations. First, it was conducted as a narrative review without a registered protocol. Although predefined inclusion criteria and dual screening were applied, the narrative format carries a risk of selection bias. Some relevant studies may have been missed, and the lack of formal quality appraisal limits the assessment of evidence strength. Second, the evidence base is uneven across regions and populations. Research from low- and middle-income countries remains limited, and key subgroups such as women, adolescents, and incarcerated individuals are underrepresented. These gaps reduce the generalizability of findings. Third, many conclusions are drawn from observational studies or modeling analyses rather than randomized controlled trials. While these sources offer important insights, their results may be influenced by confounding or assumptions and should be interpreted with caution. Finally, no quantitative synthesis was performed. As a result, the findings are descriptive and may reflect reporting trends more than comparative effectiveness. Future work, including systematic reviews and implementation trials, will be essential to strengthen the evidence base and refine prevention strategies.

## Conclusions

Preventing HIV among people who inject drugs requires more than technical interventions. It involves policy changes, consistent funding, and a shift toward accessible models of care. Expanding access to effective services such as NSPs, OAT, PrEP, and ART is essential, particularly in areas where availability remains below global targets. Addressing structural and social barriers is also necessary. This includes reducing stigma in healthcare, eliminating punitive laws, and developing services that meet the needs of women, people who are incarcerated, and LGBTQ+ individuals. Examples from Portugal and Canada suggest that legal reform combined with public health investment can reduce HIV transmission and improve health outcomes. Models that integrate HIV prevention, substance use treatment, and support services, especially when supported by peer navigation and case management, have shown potential to improve care and reduce service gaps. However, these programs are limited in many low- and middle-income countries due to restricted resources. International organizations such as the WHO and UNAIDS have called for comprehensive prevention strategies that respect human rights and respond to population needs. Aligning national policies with these recommendations and ensuring effective implementation will be important to reducing service disparities. Lowering HIV transmission among people who inject drugs will require more than expanded access to biomedical tools. Without legal reform, supportive infrastructure, and sustained investment in care systems, efforts to control the HIV epidemic will remain incomplete.
